# Interaction effect of rehabilitation initiation timing and hospitalization frequency on long-term functional outcomes after stroke in rural China: a retrospective cohort study

**DOI:** 10.3389/fneur.2025.1706724

**Published:** 2026-01-02

**Authors:** Juming Liu, Luwen Zhang, Changyu Ju, Xiping Jia, Chao Zhang, Feng Wu, Tao Qin, Qianqian Sun

**Affiliations:** 1 Xiangyang Central Hospital, Affiliated Hospital of Hubei University of Arts and Science, Xiangyang, China; 2 School of Health Services Management, Southern Medical University, Guangzhou, China; 3 School of Computer Science, Guangdong Polytechnic Normal University, Guangzhou, China; 4 Hubei University of Arts and Science, Xiangyang, China; 5 Xiangyang Key Laboratory of Rehabilitation Medicine and Rehabilitation Engineering Technology, Xiangyang, China

**Keywords:** stroke, activities of daily living, rehabilitation initiate timing, rehabilitation hospitalization frequency, rural areas

## Abstract

**Objective:**

To investigate the effects of rehabilitation-initiation timing (RIT) and rehabilitation-hospitalization frequency (RHF) on activities of daily living (ADL) evaluated at 6 months post-stroke.

**Design:**

Retrospective cohort study.

**Setting:**

Convalescent rehabilitation wards in urban and suburban areas of Xiangyang, China.

**Participants:**

A total of 275 patients with ADL impairment following acute or subacute stroke who received inpatient comprehensive rehabilitation between 2021 and 2024.

**Interventions:**

Participants underwent inpatient multidisciplinary rehabilitation—including physical therapy, occupational therapy, and individualized functional exercises—during each hospitalization, with each inpatient rehabilitation episode lasting for 3 weeks. The main exposures were the timing of rehabilitation initiation and the total number of inpatient rehabilitation episodes within the first 6 months post-stroke.

**Main outcome measures:**

The primary outcome was the change in ADL, assessed by the Barthel Index (BI), from baseline to the 6-month post-stroke follow-up.

**Results:**

At the 6-month (180-day) follow-up, the mean BI score increased by 12.59 points compared to baseline (95% CI, 5.53–19.65; *p* < 0.001). Compared to those who started rehabilitation at 61–90 days post-stroke, patients who initiated rehabilitation earlier—at 1–14, 15–30, and 31–60 days—showed greater BI improvements at 6 months, with mean differences of 15.48 (95% CI, 4.90–26.06; *p* = 0.004), 13.18 (95% CI, 3.85–22.51, *p* = 0.005), and 8.63 (95% CI, 0.40–16.86, *p* = 0.04) points, respectively. Among patients who started rehabilitation at 1–14 and 15–30 days, each additional systematic inpatient rehabilitation was associated with a further mean BI increase of 2.24 (95% CI, 0.98–5.46, *p* = 0.20) and 2.10 (95% CI, 0.87–5.07, *p* = 0.21) points, respectively, although these differences did not reach statistical significance. Subgroup analysis showed that early rehabilitation significantly improved BI in patients aged ≥65 and those with hemorrhagic stroke. Moreover, higher hospitalization frequency benefited patients with higher education and those with hemorrhagic stroke.

**Conclusion:**

Earlier initiation and greater frequency of inpatient rehabilitation were independently associated with better ADL outcomes at the 6-month mark in rural Chinese stroke survivors. Importantly, the benefit of each additional rehabilitation admission was amplified when therapy began within the first month post-stroke and diminished when initiation was delayed beyond two months, especially among patients with hemorrhagic stroke, aged ≥65 years, women, and those with higher educational attainment.

## Introduction

The acute and subacute phases, corresponding to the first few months post-stroke, represent a critical window for neurological recovery. During this period, patients exhibit heightened responsiveness to therapeutic interventions, making the analysis of rehabilitation initiation timing an essential factor for maximizing functional outcomes (1). Following a stroke, survivors frequently develop profound functional deficits and diminished activities of daily living (ADL) (2). Declining ADL in turn reduces quality of life, elevates hospitalisation and fall risk, and increases caregiver burden (3, 4). Rural residents receive poorer stroke care, and technological advances may widen this disparity (5). Achieving equitable rehabilitation—especially for rural populations—therefore remains challenging and is associated with delayed intervention and worse outcomes.

Rehabilitation is the most effective strategy for restoring ADL (6). Its benefit depends on therapy intensity,duration,and, critically, the interval between stroke onset and rehabilitation initiation (7–9). Although one study found that rural patients were less likely to receive a complete stroke-care bundle without showing worse outcomes (10), the influence of rehabilitation timing on 6-month ADL in rural settings is unclear.

Evidence supports beginning rehabilitation within 6 months after stroke (11). Among sub-acute patients, > 40 sessions yield greater ADL gains than ≤ 40 sessions (12). However, in China, community-based rehabilitation networks remain underdeveloped, with limited standardized service models, incomplete rural and community rehabilitation infrastructure, and insufficient insurance coverage for many rehabilitation programs (13, 14). As a result, stroke survivors in rural areas often require structured inpatient rehabilitation. However, due to health insurance policies that typically restrict each inpatient rehabilitation cycle to approximately three weeks, many patients must be readmitted multiple times during the crucial six-month recovery period in order to receive adequate therapy. These systemic barriers disrupt continuity of care and limit access to sustained, high-quality rehabilitation, and their impact on long-term functional recovery—particularly among rural patients—remains unclear.

Clinical and sociodemographic factors—including stroke subtype, age, sex, and education—also shape recovery trajectories (15–22). Few studies have evaluated rehabilitation initiation timing (RIT) and rehabilitation-hospitalisation frequency (RHF) together or explored their interaction with patient characteristics. This study therefore assessed their independent and combined effects on 6-month ADL, measured by the Barthel Index (BI), with subgroup analyses by stroke subtype, age, sex, and education.

## Methods

### Ethics approval

The institutional ethics committee approved the study (No. 2024–098). All participants provided written informed consent in accordance with the Declaration of Helsinki. Reporting follows STROBE guidelines.

### Study design

A retrospective cohort study was conducted at rehabilitation hospitals in urban and suburban Xiangyang, China, serving predominantly rural populations. The study assessed whether rehabilitation initiation timing and rehabilitation-hospitalization frequency within 6 months after stroke predicted change in BI score. All data collectors completed a uniform training programme. Two researchers entered the data independently, and a third auditor randomly reviewed 5% of records; discrepancies were resolved against the source documents.

### Participants

**Figure 1:**
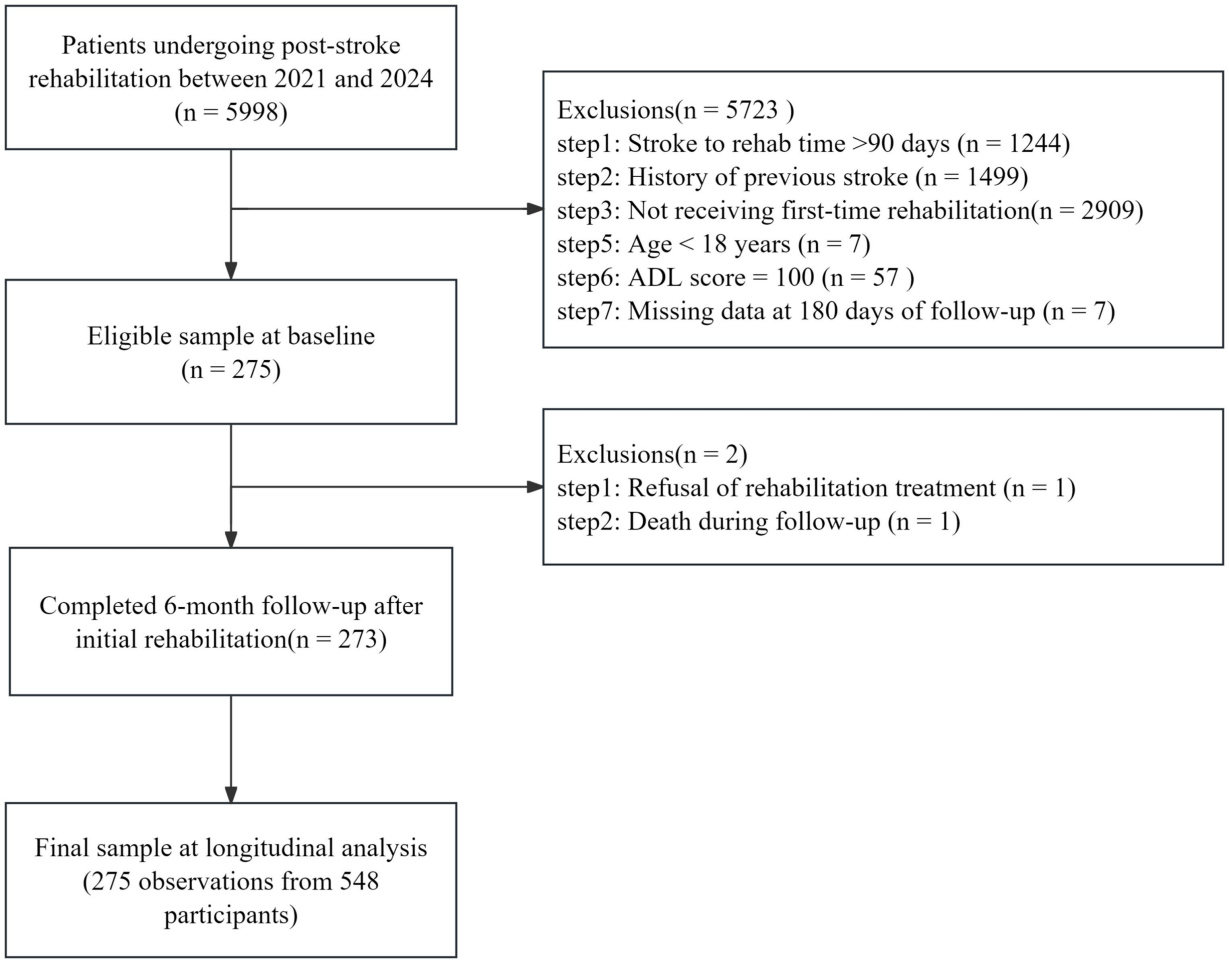
Study flowchart showing selection of participants.

All patients admitted for stroke between 1 January 2021 and 31 December 2024 were screened. Eligibility required (i)a first-ever ischaemic or haemorrhagic stroke, with lesion localization documented, verified by CT or MRI; (ii) initiation of the index rehabilitation programme within 1–90 days of stroke onset; (iii) age ≥ 18 years; (iv) first rehabilitation delivered at a participating hospital; (v) a baseline Barthel Index (BI) < 100; and (vi) sufficient cognitive and physical capacity to comply with assessment and therapy. Patients without a 180-day BI follow-up were excluded (Figure 1).

### Exposure definitions

#### Rehabilitation-initiation timing

RIT was defined as the number of calendar days from stroke onset to the first structured inpatient rehabilitation session—physiotherapy, occupational therapy, or speech-language therapy—delivered on a dedicated stroke-rehabilitation ward. For analysis, RIT was categorized a priori into four intervals: 1–14, 15–30, 31–60, and 61–90 days post-stroke.

#### Rehabilitation-hospitalization frequency

RHF was defined as the number of discrete inpatient admissions for structured stroke rehabilitation within six months after stroke onset, once the patient had reached clinical stability. Each qualifying admission includes a standardized rehabilitation assessment, stroke-specific medical management (e.g., antiplatelet or lipid-lowering therapy), and an individually tailored multimodal programme that may combine physiotherapy, occupational therapy, speech- and swallowing therapy, respiratory training, neuromuscular or low-frequency electrical stimulation, manual techniques such as joint mobilization or massage, acupuncture, short-wave diathermy, and task-oriented exercises as required. Readmissions undertaken solely for acute medical complications (e.g., pneumonia, heart failure) and all outpatient, day-hospital, or home-based sessions are excluded.

RHF varied significantly across the four rehabilitation-initiation groups (*p* < 0.001), whereas the mean length of stay per rehabilitation admission remained uniform (*p* = 0.26). Because single-admission duration was essentially constant, a higher RHF reflects a greater cumulative exposure to standardized rehabilitation that is independent of inpatient stay length. RHF was therefore selected as the principal exposure variable for subsequent dose–response analyses. Detailed values are reported in Supplementary Table S1.

### Outcomes

ADL were measured with the BI, which evaluates performance on 10 functional items; higher scores indicate greater independence. The primary outcome was the change in BI from baseline to 180 days after rehabilitation initiation.

### Covariates

Prespecified covariates were stroke subtype (ischaemic vs. haemorrhagic), sex, age, educational attainment (primary school or less vs. above primary), hypertension, diabetes mellitus, hyperlipidaemia, coronary heart disease, prior stroke-related surgery, venous thromboembolism risk score, and current smoking or alcohol use (yes vs. no).

### Statistical analysis

All computations were performed with R (version 4.3.1; R Foundation for Statistical Computing, Vienna, Austria). Continuous variables are expressed as mean ± standard deviation (SD) or median (interquartile range) where appropriate, whereas categorical variables are reported as counts and percentages. Baseline characteristics were compared with χ^2^ or Fisher’s exact tests for categorical data and with one-way analysis of variance or Kruskal–Wallis tests for continuous data, according to distributional assumptions. Two-sided *p* values < 0.05 were regarded as statistically significant. The impact of rehabilitation-initiation timing and RHF on 6-month change in BI was examined with linear mixed-effects models that included a random intercept for each patient. RIT was modelled as a four-level factor (1–14, 15–30, 31–60, and 61–90 days after stroke onset), while RHF—defined as the number of qualifying rehabilitation admissions within the first six months—was entered as a continuous term. Models were adjusted a priori for the demographic and clinical covariates listed above. Pre-specified subgroup and sensitivity analyses are described in the Supplementary material.

## Results

### Participant characteristics

Table 1 presents baseline data for the 275 participants, stratified by RIT. Baseline BI scores were comparable across timing groups (*p* = 0.52), and no significant differences emerged in sex, age, body-mass index, educational level, lifestyle behaviours, or major comorbidities (all *p* > 0.05). By contrast, stroke subtype and the prevalence of prior stroke-related surgery differed significantly among the four RIT groups (*p* = 0.01; *p* = 0.003).

**Table 1:** Demographics and clinical characteristics.

Characteristics	Group 1 (1–14) days	Group 2 (15–30) days	Group 3 (31–60) days	Group 4 (61–90) days	*p*-value
Stroke type, *n* (%)					0.01
Ischemic stroke	37 (13.45%)	39 (14.18%)	33 (12.00%)	14 (5.09%)	
Hemorrhagic stroke	10 (3.64%)	53 (19.27%)	48 (17.45%)	27 (9.82%)	
Other	0 (0.00%)	5 (1.82%)	6 (2.18%)	3 (1.09%)	
Sex, *n* (%)					0.45
Male	35(12.73)	70(25.45)	55(20.00)	29(10.55)	
Female	12(4.36)	27(9.82)	32(11.64%)	15(5.45%)	
Age, Mean ± SD	63.13 ± 5.11	61.06 ± 3.00	55.70 ± 2.80	63.25 ± 5.44	0.85
Educational level, *n* (%)					0.70
Primary	8(2.91%)	22(8.00%)	19(6.91%)	12(4.36%)	
Above primary	39(14.18%)	75(27.27%)	68(24.73%)	32(11.64%)	
Smoking status, *n* (%)					0.92
No	30(10.91%)	66(24.00%)	59(21.45%)	31(11.27%)	
Yes	17(6.18%)	31(11.27%)	28(10.18%)	13(4.73%)	
Drinking status, *n* (%)					0.56
No	32(11.64%)	61(22.18%)	62(22.55%)	32(11.64%)	
Yes	15(5.45%)	36(13.09%)	25(9.09%)	12(4.36%)	
Surgical history, *n* (%)					0.003
No	29(10.55%)	37(13.45%)	50(18.18%)	15(5.45%)	
Yes	18(6.55%)	60(21.82%)	37(13.45%)	29(10.55%)	
Hypertension, *n* (%)					0.5
No	7 (2.55%)	18 (6.55%)	21 (7.64%)	11 (4.00%)	
Yes	40 (14.55%)	79 (28.73%)	66 (24.00%)	33 (12.00%)	
Hyperlipidemia, *n* (%)					0.15
No	44 (16.00%)	86 (31.27%)	83 (30.18%)	43 (15.64%)	
Yes	3 (1.09%)	11 (4.00%)	4 (1.45%)	1 (0.36%)	
Diabetes, *n* (%)					0.31
No	31 (11.27%)	77 (28.00%)	67 (24.36%)	35 (12.73%)	
Yes	16 (5.82%)	20 (7.27%)	20 (7.27%)	9 (3.27%)	
Coronary heart disease, *n* (%)					0.86
No	40 (14.55%)	86 (31.27%)	76 (27.64%)	37 (13.45%)	
Yes	7 (2.55%)	11 (4.00%)	11 (4.00%)	7 (2.55%)	
VTE scores, Mean ± SD	1.38 ± 0.32	2.50 ± 0.52	2.26 ± 0.46	2.00 ± 0.87	0.43
BI scores at baseline, Mean ± SD	35.63 ± 13.08	39.81 ± 7.23	30.96 ± 3.56	33.13 ± 8.34	0.52
BI scores at 180-day follow-up, Mean ± SD	65.02 ± 4.10	55.69 ± 2.37	52.82 ± 2.56	47.04 ± 3.75	<0.01

VTE, venous thromboembolism; BI, Barthel Index.

### Impact of RIT on ADL recovery

After multivariable adjustment, baseline BI scores did not differ between any early-initiation group (RIT 1–14 d, 15–30 d, or 31–60 d) and the late-initiation reference group (61–90 d; all *p* > 0.05; Table 2). By contrast, earlier RIT was associated with significantly greater functional gains at 180 days. Initiating rehabilitation within 1–14 days produced the largest benefit (*β* = 15.48, 95% CI 4.90–26.06; *p* = 0.004), followed by 15–30 days (*β* = 13.18, 95% CI 3.85–22.51; *p* = 0.005) and 31–60 days (*β* = 8.63, 95% CI 0.40–16.86; *p* = 0.040) compared with the reference group. The overall time effect indicated a mean improvement of 12.59 BI points across the cohort during follow-up (95% CI 5.53–19.65; *p* < 0.001).

**Table 2:** Association between RIT and 6-month change in BI.

Group Comparison	Standardized β-coefficient	SE	95% CI	*P*-value
Baseline				
Group 1 vs. group 4	−0.50	4.28	−8.89, 7.89	0.91
Group 2 vs. group 4	−2.89	3.87	−10.48,4.70	0.50
Group 3 vs. group 4	−1.17	3.07	−7.19, 4.85	0.70
180-day follow-up				
Group 1 vs. group 4	15.48	5.40	4.90, 26.06	0.004
Group 2 vs. group 4	13.18	4.76	3.85, 22.51	0.005
Group 3 vs. group 4	8.63	4.20	0.40, 16.86	0.04
Time effect	12.59	3.60	5.53, 19.65	<0.001

*A multiple-exposure model adjusted for age, sex, educational level, clinical history variables, and lifestyle factors, with mutual adjustment for the initial rehabilitation initiate timing group and time effects in stroke patients.

### Interaction between RHF and RIT on ADL recovery 

The linear mixed-effects model revealed a positive dose–response between RHF and 6-month BI change: each additional qualifying admission was associated with a 1.47-point increment in BI (*β* = 1.47, 95% CI 0.27–2.67; *p* = 0.010; Table 3). When stratified by RIT, the magnitude of this effect varied: relative to the late-initiation reference group (61–90 days), each extra admission in the 1-14-day and 15-30-day cohorts conferred larger gains of 2.24 and 2.10 BI points, respectively, whereas the 31-60-day cohort showed a modest 0.49-point increase.

**Table 3:** Interaction between RHF and RIT on 6-month BI change.

Group comparison	Standardized β-coefficient	SE	95% CI	*P*-value
RHF (per admission)	1.47	0.61	0.27, 2.67	0.01
Group comparison				
Group 4 ref. (reference)	-	-	-	-
Group1 vs. group 4	2.24	1.64	−0.98, 5.46	0.20
Group 2 vs. group 4	2.10	1.51	−0.87, 5.07	0.21
Group 3 vs. group 4	0.49	1.43	−2.32, 3.30	0.85

*A multiple-exposure model adjusted for age, sex, educational level, time effects, clinical history variables, and lifestyle factors, with mutual adjustment for both the RIT and RHF. RIT, rehabilitation initiation timing; RHF, rehabilitation-hospitalisation frequency.

### Subgroup analyses

**Figure 2:**
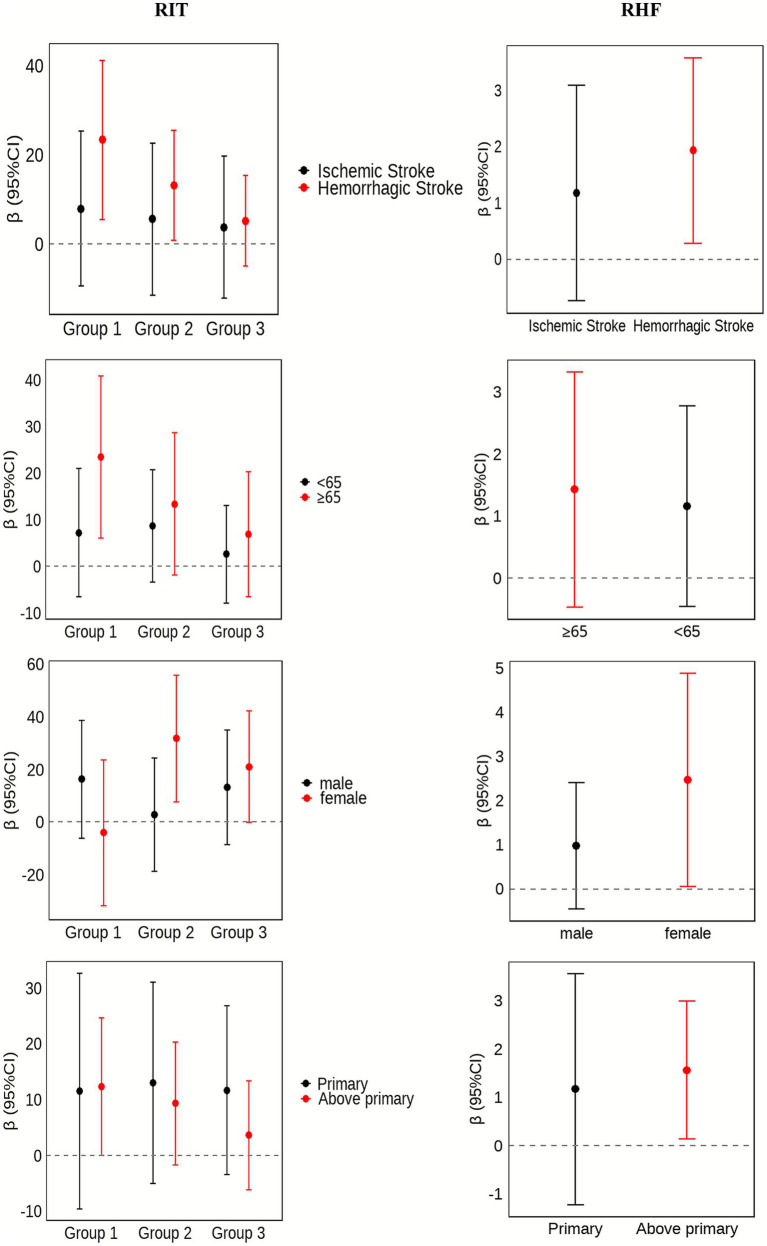
Sub group effects of RIT and RHF on 6-month change in BI, stratified by stroke subtype, sex, educational attainment, and age. RIT, rehabilitation initiation timing; RHF, rehabilitation-hospitalisation frequency.

To investigate whether the effects of RIT and RHF differed across key patient populations, particularly given the known differences in recovery trajectories between ischemic and hemorrhagic strokes, pre-specified subgroup analyses were conducted. Figure 2 summarises the subgroup findings. Early RIT was associated with significantly larger 6-month BI gains in patients aged ≥ 65 years (*β* = 20.97, 95% CI 3.62–38.31; *p* = 0.02) and in those with haemorrhagic stroke (*β* = 22.05, 95% CI 4.59–39.51; *p* = 0.01); no benefit was observed in the remaining strata. For RHF, each additional rehabilitation admission yielded significant improvements in participants with higher educational attainment (*β* = 1.59, 95% CI 0.18–3.01; *p* = 0.03) and in the hemorrhagic stroke subgroup (*β* = 1.89, 95% CI 0.28–3.49; *p* = 0.02). No significant RHF related gains were detected in the other subgroups. Full numerical details are provided in Supplementary Tables S2, S3.

### Sensitivity analyses

The sensitivity analysis confirmed the primary findings, the direction and magnitude of the effects were essentially unchanged. Earlier RIT and higher RHF each remained independently associated with greater 6-month gains in BI, underscoring the robustness of the results (Supplementary Tables S4, S5).

## Discussion

To the best of the authors’ knowledge, this study is the first real-world cohort to quantify both RIT and RHF as drivers of long-term ADL after stroke in China. The results show that starting structured rehabilitation earlier—and repeating it more often—independently predicts larger 6-month gains in BI. Subgroup analyses highlight clinically meaningful heterogeneity by stroke subtype, age, sex, and educational attainment. Notably, many patients in the study cohort, particularly from rural areas, began rehabilitation more than 30 days post-stroke, a delay likely driven by limited health literacy and restricted access to specialized care. Collectively, these findings offer actionable evidence for refining post-stroke rehabilitation policies and tailoring intervention schedules to maximize functional recovery.

This study demonstrated that initiating rehabilitation within 1–90 days post-stroke improves ADL at 6 months, with the largest gains when therapy begins in the first 14 days. This underscores how critical prompt rehabilitation is for functional recovery. Supporting these findings, a nationwide Japanese cohort showed that rehabilitation initiated within 3 days of hospitalization significantly improved ADL at discharge (8), and other retrospective work has likewise identified early therapy and longer inpatient exposure as key predictors of ADL improvement (9). Mechanistically, early rehabilitation coincides with the peak of post-ischaemic neuroplasticity, fostering network re-organization, synaptogenesis, and restoration of cortical excitability (16–18). These processes are most active soon after stroke, explaining the larger ADL observed in this study. Patients who start therapy within 1–30 days may still be in a phase of cerebral-oedema resolution and active neuro-regeneration (19, 20); as oedema subsides and neuro-inflammation diminishes, the micro-environment becomes more conducive to repair (21, 22). By contrast, delaying rehabilitation (RIT 31–90 days) risks missing this plasticity window. Early therapy also curbs secondary complications—deep-vein thrombosis, infections, and secondary brain injury —which further supports recovery (23, 24).

The analysis also confirmed the positive effect of RHF on ADL recovery. After adjustment for all demographic and clinical covariates, each additional rehabilitation admission was associated with a mean gain of 1.47 BI points. When the RHF effect was stratified by rehabilitation-initiation timing (RIT), the greatest marginal benefit was seen in the earliest-initiation groups: relative to the 61–90-day reference group, every extra admission raised the 6-month BI by 2.24 points when rehabilitation began within 1–14 days and by 2.10 points when it began within 15–30 days, whereas the corresponding increase was 0.49 points for initiation at 31–60 days. Although some comparisons did not reach statistical significance—likely a consequence of limited sample size—the directional consistency and effect sizes are clinically meaningful for stroke rehabilitation (25).

These findings reinforce earlier work emphasising the central role of rehabilitation “dose” in post-stroke recovery. A Korean cohort study, for example, showed that > 40 rehabilitation sessions within six months improved ADL and reduced all-cause mortality (12), while a meta-analysis by Kwakkel et al. (26) reported significant gains in ADL and motor function for each additional hour of therapy. Repeated admissions likely provide sustained sensorimotor stimulation that facilitates neural remodeling and synaptic regeneration (27). In China, insurance regulations restrict prolonged inpatient rehabilitation, resulting in multiple short-cycle admissions that deliver intensive, therapist-guided interventions. Consequently, RHF approximates the cumulative volume of rehabilitation actually received. For rural patients with limited access to continuous outpatient services, increasing the number of such short admissions may partially offset delays in initiation and help close the urban–rural gap in post-stroke care.

Subgroup analyses uncovered clinically meaningful heterogeneity. Early RIT yielded the largest BI gains in patients with haemorrhagic stroke and those aged ≥ 65 years, whereas the effect of RHF was most pronounced among patients with hemorrhagic stroke and higher educational attainment, with a borderline trend observed in women. The heightened responsiveness of hemorrhagic stroke patients aligns with Korean registry data showing reduced mortality only in this subgroup (12). likely reflecting reversible mass-effect resolution and preserved neuroplastic potential likely reflecting reversible mass-effect resolution and preserved neuroplastic potential (28, 29). Older adults experienced greater functional improvements, perhaps owing to lower baseline independence and a larger absolute capacity for recovery, as suggested by neuroimaging studies (30, 31). Women achieved more pronounced BI increases, potentially because of higher baseline frailty and sociocultural factors influencing rehabilitation engagement (32, 33). Finally, higher education was associated with better outcomes—likely through improved health literacy and adherence—consistent with studies on stroke awareness and participation (34, 35). Conversely, limited education and health literacy in rural populations may impede effective rehabilitation uptake, underscoring the need for targeted stroke-education programmes and community-based support.

The strengths of this study include its longitudinal design and the first quantitative analysis of both RIT and RHF on ADL recovery in stroke patients. By examining their interaction, the study’s findings highlight the importance of early and frequent inpatient rehabilitation within the first six months post-stroke and provide new clinical evidence for optimizing rehabilitation strategies. Importantly, RHF is especially relevant in the Chinese context, where insurance constraints limit prolonged stays and rural patients have scarce outpatient options—multiple short-cycle admissions therefore offer a practical means to deliver cumulative therapy “dose.” The use of linear mixed-effects models ensured rigorous control of confounders, supporting the reliability of the results, and subgroup analyses revealed heterogeneity in response, underscoring the need for personalized rehabilitation plans tailored to patient characteristics.

However, several limitations should be acknowledged. First, this study primarily assessed the relationship between RIT, RHF, and ADL improvement, without further differentiating among specific types of rehabilitation interventions (e.g., physical therapy, occupational therapy). Second, the model used in this study did not account for other critical real-world factors that can disrupt the rehabilitation process. For instance, progress can be delayed or interrupted by medical complications—some of which may be unrelated to the primary stroke pathology (36). Moreover, variables such as the total number of rehabilitation days lost due to illness or other reasons, and the patient’s psychological state (e.g., depression, anxiety, or motivational deficits), are known to significantly influence functional outcomes. Although this study did not use specific scales to assess these factors, their impact on recovery and autonomy is undeniable and represents an important area for future, more granular research. Third, the study’s findings require validation in larger-scale studies.

## Conclusion

Earlier RIT and higher RHF are independently linked to greater six-month ADL improvements after stroke, with the strongest effects observed in patients starting rehabilitation within 30 days and undergoing more frequent admissions. These findings are especially relevant in rural China, where limited community rehabilitation and short insurance-covered stays necessitate repeated inpatient rehabilitation. By quantifying these effects, this study highlights the importance of timely, sustained rehabilitation and underscores the need to strengthen coverage and expand community services to optimize functional recovery.

## Data Availability

The raw data supporting the conclusions of this article will be made available by the authors, without undue reservation.
