# Supplementary material for: Interaction effect of rehabilitation initiation timing and hospitalization frequency on long-term functional outcomes after stroke in rural China: a retrospective cohort study
Source: Front Neurol. 2026 Jan 2;16:1706724. doi: 10.3389/fneur.2025.1706724 (PMC12807886; doi:10.3389/fneur.2025.1706724)
Supplement: Supplementary file 1 [file Table_1.docx]

**Supplemental Materials**

**Data Collection**

This included clinical features, demographic characteristics, comorbidities, adverse lifestyle habits, and the MBI scores at admission.

(1) Clinical Features: Stroke sub-type, stroke onset days, hospital frequency;

(2) Demographic Characteristics: Included gender, age, and education level. The education level was categorized into three levels: primary (primary school and below), secondary (junior high and high school), and higher (college and above);

(3) Comorbidities: Presence of hypertension, diabetes, hyperlipidemia, coronary heart disease, VTE scores, history of previous stroke surgeries;

(4) Adverse Lifestyle Habits: Smoking and alcohol consumption;

(5) MBI score: In this study, the Barthel Index (BI) was employed to assess the ADL of stroke patients. This scale comprises 10 items: bowel control, bladder control, eating, dressing, toileting, personal hygiene, bathing oneself, transferring, walking, and climbing stairs. Scores are assigned as follows: 10 for complete independence, 8 for minimal assistance, 5 for moderate assistance, 2 for maximal assistance, and 0 for complete dependence. The maximum score is 100, with higher scores reflecting greater independent living ability.

**Detailed Statistical Analysis**

All statistical analyses were performed using R software (R Foundation for Statistical Computing, Vienna, Austria). Continuous variables were summarized using means and standard deviations, whereas categorical variables were presented as counts and percentages. Baseline characteristics across the four rehabilitation timing groups (1–14 days, 15–30 days, 31–60 days, and 61–90 days post-stroke onset) were compared using appropriate statistical tests. For categorical variables, the chi-square test or Fisher’s exact test was applied, while for continuous variables, one-way analysis of variance (ANOVA) or the Kruskal–Wallis test was used, depending on the normality of the data distribution.The primary outcome was the change in the BI score from baseline to six months post-stroke. To accommodate repeated measures and account for intra-individual correlations, linear mixed-effects models (LMM) were implemented using the lme4 and lmerTest packages in R, with subject identifier included as a random effect. Fixed effects in the models comprised rehabilitation initiation timing group, follow-up time points (baseline and 6 months), hospitalization frequency within six months, and a range of covariates: age, gender, education level, marital status, stroke subtype, stroke stage, comorbidities (hypertension, hyperlipidemia, diabetes, coronary heart disease, and venous thromboembolism), lifestyle factors (smoking and alcohol consumption), and baseline MBI score. Interaction terms were incorporated to explore whether the effect of rehabilitation timing on MBI improvement varied by follow-up time and to determine if the relationship between hospitalization frequency and MBI change differed across timing groups. Subgroup analyses were conducted by stratifying patients according to stroke subtype, gender, age group (≥65 vs. <65 years), and education level, employing similar LMM approaches and including relevant interaction terms to test for potential effect modification.

**Sensitivity Analysis Methods**

To confirm the robustness of the primary findings, sensitivity analyses were conducted using two distinct multiple-exposure models. Model 1 was designed to evaluate the effect of rehabilitation initiation timing on ADL at six months, adjusting for age, gender, and education level, thereby assessing the interplay between timing and time effects. Model 2 built upon Model 1 by additionally adjusting for comorbidities (hypertension, hyperlipidemia, diabetes, coronary heart disease) and lifestyle factors (smoking and alcohol consumption) to address potential confounding by chronic conditions and behaviors.

To further investigate the interaction between rehabilitation hospitalization frequency and initiation timing on MBI scores, Model 3 included an interaction term between these two variables as the primary exposure, with adjustments for age, gender, education level, and time effects. Model 4 extended this by incorporating additional confounders, including hypertension, hyperlipidemia, diabetes, coronary heart disease, venous thromboembolism, smoking, and alcohol consumption. All sensitivity analyses utilized LMM, with subjects as random effects to account for repeated measures. Fixed effects encompassed all pertinent covariates, enabling a comprehensive evaluation of the consistency and reliability of the results across varying adjustment strategies.

**Table S1. Distribution of rehabilitation-hospitalization frequency and mean length-of-stay per admission across rehabilitation-initiation timing groups**

| Characteristics | Group 1  (1-14)days | Group 2  (15-30)days | Group 3  (31-60)days | Group 4  (61-90)days | *P*-value |
| --- | --- | --- | --- | --- | --- |
| Rehabilitation-hospitalization frequency（times），mean ± SD | 2.63±0.29 | 2.97±0.19 | 3.03±0.16 | 2.26±0.16 | < 0.001 |
| Mean length of stay per rehabilitation admission (days), mean ± SD | 20.90±0.83 | 21.10±0.53 | 21.20±0.58 | 20.85±0.90 | 0.26 |

**P*-value were obtained as follows: rehabilitation-hospitalization frequency (non-normal distribution) by Kruskal–Wallis test; mean length of stay (approximately normal, homoscedastic) by one-way ANOVA.

**Table S2. Subgroup Analysis of the rehabilitation initiation timing on BI scores by stroke type, gender, education level, and age**

| Group | Subgroup | Estimate *β* | 95% CI | P-value |
| --- | --- | --- | --- | --- |
| Group1 | ≥65 | 20.97 | 3.62 to 38.31 | 0.02 |
| Group2 | ≥65 | 10.75 | -4.49 to 26.00 | 0.17 |
| Group3 | ≥65 | 4.25 | -9.18 to 17.68 | 0.53 |
| Group1 | <65 | 7.49 | -5.93 to 20.90 | 0.27 |
| Group2 | <65 | 8.76 | -3.08 to 20.60 | 0.15 |
| Group3 | <65 | 2.74 | -7.53 to 13.00 | 0.60 |
| Group1 | Male | 22.06 | -0.23 to 44.35 | 0.06 |
| Group2 | Male | 8.62 | -12.81 to 30.04 | 0.37 |
| Group3 | Male | 18.53 | -3.13 to 40.18 | 0.09 |
| Group1 | Female | -9.51 | -36.69 to 17.68 | 0.49 |
| Group2 | Female | 26.12 | 2.53 to 49.70 | 0.03 |
| Group3 | Female | 15.13 | -5.60 to 35.86 | 0.15 |
| Group1 | Primary | 10.84 | -1.33 to 23.01 | 0.09 |
| Group2 | Primary | 7.75 | -3.12 to 18.61 | 0.19 |
| Group3 | Primary | 1.95 | -7.68 to 11.58 | 0.72 |
| Group1 | Above primary | 14.08 | -5.87 to 34.04 | 0.16 |
| Group2 | Above primary | 15.44 | -1.57 to 32.46 | 0.09 |
| Group3 | Above primary | 14.07 | -0.19 to 28.34 | 0.05 |
| Group1 | Ischemic Stroke | 10.15 | -7.17 to 27.46 | 0.25 |
| Group2 | Ischemic Stroke | 7.75 | -9.23 to 24.73 | 0.37 |
| Group3 | Ischemic Stroke | 5.94 | -9.88 to 21.76 | 0.46 |
| Group1 | Hemorrhagic Stroke | 22.05 | 4.59 to 39.51 | 0.01 |
| Group2 | Hemorrhagic Stroke | 11.78 | -0.24 to 23.81 | 0.05 |
| Group3 | Hemorrhagic Stroke | 3.75 | -6.16 to 13.65 | 0.46 |

**Table S3. Subgroup Analysis of the rehabilitation hospitalization frequency on BI scores by stroke type, gender, education level, and age**

| Subgroup | Estimate *β* | 95% CI | P-value |
| --- | --- | --- | --- |
| Male | 1.06 | -0.36 to 2.48 | 0.17 |
| Female | 2.14 | -0.19 to 4.48 | 0.07 |
| ≥65 | 1.48 | -0.43 to 3.38 | 0.13 |
| <65 | 1.16 | -0.42 to 2.75 | 0.15 |
| Primary | 1.06 | -1.20 to 3.33 | 0.36 |
| Above primary | 1.59 | 0.18 to 3.01 | 0.03 |
| Ischemic Stroke | 1.21 | -0.69 to 3.12 | 0.22 |
| Hemorrhagic Stroke | 1.89 | 0.28 to 3.49 | 0.02 |

**Table S4. Sensitivity analysis of the impact of rehabilitation initiation timing on ADL scores: baseline and 6-Month follow-up comparisons**

| Group Comparison | Model 1 | | Model 2 | |
| --- | --- | --- | --- | --- |
|  | *β* (95% CI) | *P-*Value | *β* (95% CI) | *P-*Value |
| Baseline |  |  |  |  |
| Group1 vs group 4 | 4.24 (-4.87, 13.36) | 0.36 | 1.94 (-3.72, 7.61) | 0.54 |
| Group 2 vs group 4 | -1.43 (-9.31, 6.45) | 0.72 | 0.41 (-4.47, 5.29) | 0.87 |
| Group 3 vs group 4 | 1.25 (-6.76, 9.26) | 0.76 | 1.18 (-3.85, 6.21) | 0.64 |
| 180-day follow-up |  |  |  |  |
| Group1 vs group 4 | 16.47 (8.43, 24.51) | <0.001 | 16.29(8.42, 24.16) | <0.001 |
| Group 2 vs group 4 | 13.10 (6.14, 19.06) | <0.001 | 12.67 (5.64, 19.69) | <0.001 |
| Group 3 vs group 4 | 7.72 (0.17, 15.27) | 0.04 | 7.37 (0.09, 14.65) | 0.04 |
| Time effect | 18.90(12.78, 25.03) | <0.001 | 17.41(11.18, 23.64) | <0.001 |

Model 1 : A multiple-exposure model adjusted for age, gender, and education level, with mutual adjustment for the initial rehabilitation time window grouping of stroke patients and time effect.

Model 2 : A multiple-exposure model adjusted for the covariates in Model 1, with additional adjustment for hypertension, hyperlipidemia, diabetes, coronary heart disease, smoking status, and alcohol status.

**Table S5. Sensitivity analysis of impact of the rehabilitation hospitalization frequency and grouping interaction on ADL scores**

| Group Comparison | Model 3 | | Model 4 | |
| --- | --- | --- | --- | --- |
|  | *β* (95% CI) | *P-*Value | *β* (95% CI) | *P-*Value |
| Hospitalization frequency | 0.04 (-1.39, 1.32) | 0.95 | 0.73 (0.33, 1.79) | 0.01 |
| Group 4 ref | 2.35 (-5.22, 0.52) | 0.10 | 1.20 (-3.64, 1.23) | 0.33 |
| Group1 vs group 4 | 3.83 (0.79, 6.87) | 0.01 | 3.58 (0.92, 6.24) | 0.01 |
| Group 2 vs group 4 | 2.63 (-0.09, 5.36) | 0.06 | 2.16 (-0.24, 4.56) | 0.07 |
| Group 3 vs group 4 | 1.25 (-1.52, 4.02) | 0.37 | 0.87 (-1.58, 3.32) | 0.48 |

Model 3 : A multiple-exposure model adjusted for age, gender, education level,and time effect with mutual adjustment for the initial rehabilitation time window grouping of stroke patients and the rehabilitation hospitalization frequency .

Model 4 : A multiple-exposure model adjusted for the covariates in Model 1, with additional adjustment for hypertension, hyperlipidemia, diabetes, coronary heart disease, smoking status, and alcohol status.
